# Resolving Heterophile Antibody Interference in Viral Serology Using Blocking Tubes

**DOI:** 10.1002/mbo3.70098

**Published:** 2025-10-27

**Authors:** Junhyup Song, Ilyoub Jeong, Sinyoung Kim, Younhee Park

**Affiliations:** ^1^ Departments of Laboratory Medicine, Severance Hospital Yonsei University College of Medicine Seoul Republic of Korea

**Keywords:** analytical interference, diagnostic virology, Ebstein Barr virus, heterophile antibody, immunoassay

## Abstract

Despite their clinical utility, immunoassays are susceptible to various types of interference. In this study, we aimed to assess the extent of interference from heterophile antibodies in routine clinical tests and evaluate the effectiveness of heterophile blocking tubes (HBT) in mitigating such interference. We collected 185 residual serum samples that tested positive or equivocal in at least one IgM assay for Epstein–Barr virus (EBV), viral capsid antigen (VCA), herpes simplex virus (HSV), varicella‐zoster virus, cytomegalovirus (CMV), rubella virus, or *Toxoplasma gondii*. These samples were obtained from the clinical laboratory of a tertiary teaching hospital in Korea and tested between May and July 2024. For each sample, complete IgM and IgG results for all six pathogens were obtained by performing any missing assays. Each sample was then pretreated with HBT and reanalyzed, and the assay results were compared with those of untreated samples. HBT pretreatment significantly reduced both reactivity levels (e.g., EBV VCA: 32.2 ± 35.8 U/mL to 12.8 ± 15.6 U/mL; HSV: 1.4 ± 1.0 index to 0.6 ± 0.4 index) and positivity rates (EBV VCA: 38/185 [20.5%] to 5/185 [2.7%]; HSV: 92/185 [49.7%] to 5/185 [2.7%]). These changes notably altered the clinical interpretation of the EBV status, reclassifying the 46 patients previously identified as having primary EBV infection. Our findings indicate a high prevalence of heterophile antibody interference in routine IgM testing for common viruses. HBT pretreatment effectively eliminated this interference and may be valuable for resolving discrepancies in clinical laboratory results.

## Graphical abstract

In this study, we examined heterophile antibody interference in viral serology and evaluated the effectiveness of blocking tubes. Heterophile antibodies markedly increased false‐positive EBV VCA IgM results, leading to apparent active infection; pretreatment with blocking tubes resolved much of this interference. It also converted numerous HSV IgM cases from positive to negative, highlighting the broad applicability of heterophile blocking tube.

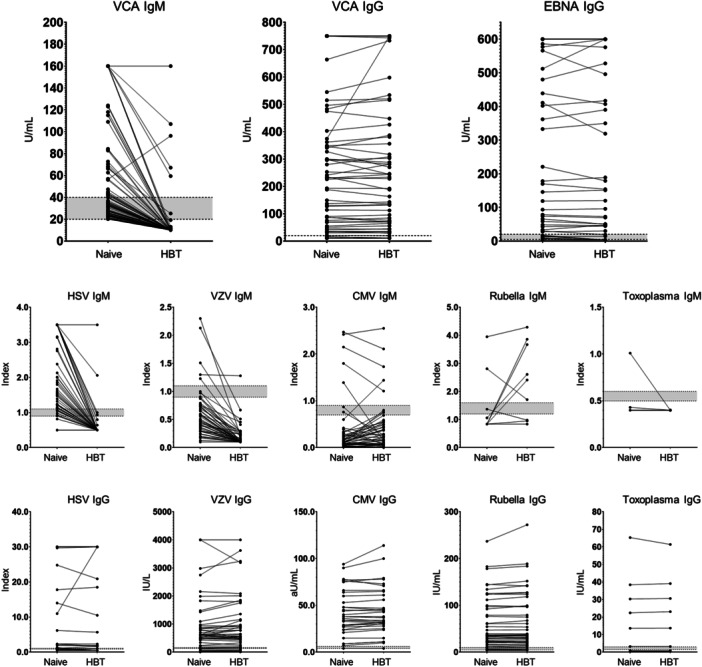

**Abbreviations**
CMV: cytomegalovirus
EBNA: Epstein–Barr nuclear antigen
EBV: Epstein–Barr Virus
HBT: heterophile blocking tube
HSV: herpes simplex virus
IgG: immunoglobulin G
IgM: immunoglobulin M
PCR: polymerase chain reaction
PTLD: post‐transplant lymphoproliferative disorder
VCA: viral capsid antigen
VZV: varicella‐zoster virus

## 1 Introduction

Although the focus of viral identification in clinical virology has increasingly shifted toward nucleic acid‐based methods, the detection of virus‐specific antibodies remains vital for diagnosing and monitoring many viral infections (Schmitz et al. 2022). Serological assays offer advantages such as lower cost and the ability to determine host immune status, making them useful first‐line screening tools for common viral infections, including Epstein–Barr virus (EBV), herpes simplex virus (HSV), cytomegalovirus (CMV), varicella‐zoster virus (VZV), and hepatitis viruses.

However, immunoassays are prone to various forms of interference, such as cross‐reactivity with other substances, interfering alloantibodies or autoantibodies, and reagent components such as biotin (Ward et al. 2017; Avery 2019). In particular, immunoglobulin M (IgM) assays for diagnosing acute infections are especially vulnerable to false‐positive results, which can complicate clinical interpretation (Landry 2016). Such spurious reactivity has been documented in assays for EBV, CMV, human immunodeficiency virus (HIV), measles, rubella, and hepatitis A and E viruses (Ghinoiu et al. 2009; Hyams et al. 2014; Jenkerson et al. 1995).

A major cause of this interference is the presence of heterophile antibodies, which are naturally occurring human antibodies that bind nonspecifically to animal‐derived monoclonal antibodies used in immunoassays (Bolstad et al. 2013; Boscato and Stuart 1988). This interference, particularly affecting sandwich immunoassays, typically results in false‐positive results, although it has also been reported in some competitive assays (Zaidi and Cowell 2010; Brugts et al. 2009). Throughout the 21st century, as many clinical laboratories have adopted commercial immunoassays compatible with automated platforms to accommodate high‐throughput testing, interference from heterophile antibodies has continued to be reported (Preissner et al. 2005; Serei et al. 2019; Lavoie et al. 2018).

To address this issue, assay manufacturers have developed and implemented several countermeasures, such as incorporating trace amounts of animal serum, immunoglobulins, or antibody fragments derived from the same species as the assay antibodies into reagents (Bolstad et al. 2013). Nonetheless, such interference persists even in advanced immunoassays. Clinical laboratories often employ additional strategies to resolve suspicious assay results (Levinson and Miller 2002), including the use of heterophile blocking tubes (HBT), which offer a practical and accessible solution as it is commercially available and can be readily integrated into the assay workflow through a brief pre‐measurement incubation of the specimen in the blocking tube.

In this study, we investigated samples that tested positive in serological assays for acute viral infections. Suspected false‐positive cases were retested following HBT pretreatment to evaluate the prevalence of heterophile antibody interference in clinical practice and assess the effectiveness of HBT in mitigating such interference.

## 2 Materials and Methods

### 2.1 Study Overview

The aim of our study was to investigate the potential for false‐positivity among clinically important viruses in specimens with suspected active infection and to determine whether such false‐positive cases are associated with the presence of heterophile antibodies, and if so, whether they can be effectively eliminated by treatment with HBT. Therefore, we first collected all samples for which a clinical serology test order had been placed for one or more of EBV VCA, HSV, VZV, CMV, rubella, or *Toxplasma gondii* and tested positive or equivocal for IgM. Because most samples had not originally been tested for all six pathogens together, each specimen was supplemented with the missing IgG/IgM tests so that complete results for EBV VCA, HSV, VZV, CMV, rubella, and Toxplasma gondii were available for research purpose. As a result, a data set of 185 samples with complete IgG/IgM results for the six pathogens was obtained, and these samples were subjected to additional assays to evaluate the presence of heterophile antibodies and the effect of HBT treatment.

### 2.2 Samples

Samples were collected at the clinical laboratory of a tertiary teaching hospital in northwest Seoul, Korea. Samples were included if they tested positive or equivocal for IgM in any serologic assay for EBV VCA or other common viral or parasitic infections, such as HSV, VZV, CMV, rubella, or *Toxoplasma gondii*. A total of 185 serum samples that met this criterion were sequentially collected and tested between May and July 2024.

### 2.3 Ethical Approval

The study protocol was reviewed and approved by the Institutional Review Board of Severance Hospital, Seoul, Korea (IRB No. 1‐2024‐0017). The need for informed consent was waived because of the study's retrospective nature and minimal risk, provided that patient confidentiality was strictly protected.

### 2.4 Assays

The assays were performed using multiple automated immunoassay platforms, reflecting the routine practice of our clinical laboratory: the Liaison XL (DiaSorin SpA, Saluggia, Italy) for EBV (VCA IgM, VCA IgG, EBNA IgG), HSV (IgM, IgG), and VZV (IgM, IgG) assays; the VIDAS system (BioMérieux, Marcy‐l'Etoile, France) for CMV (IgM, IgG); and the Architect i2000 (Abbott Diagnostics, Abbott Park, IL, USA) for rubella (IgM, IgG) and *Toxoplasma gondii* (IgM, IgG) (Cutoff values are summarized in Table 1). These platform allocations primarily reflect the testing capacity and workflow of our laboratory, where certain assays are restricted to specific platforms, while others are distributed across platforms to optimize throughput.

**Table 1:** Manufacturer‐recommended cutoffs for each assay on the three automated immunoanalyzers.

	Liaison							VIDAS		Architect			
Assay principle Target virus Sample volume, μL Isotype Reporting unit	CLIA							ELFA		CMIA			
	EBV			HSV		VZV		CMV		Rubella		Toxoplasma	
	200			200		200		200		100		100	
	VCA IgM	VCA IgG	EBNA IgG	IgM	IgG	IgM	IgG	IgM	IgG	IgM	IgG	IgM	IgG
	U/mL	U/mL	U/mL	Index	Index	Index	IU/L	Index	aU/mL	Index	IU/mL	Index	IU/mL
Interpretation													
Reactive	< 20.0	< 20.0	< 5.0	< 0.9	< 0.9	< 0.9	< 135.0	< 0.7	< 4.0	< 1.2	< 5.0	< 0.5	< 1.6
Equivocal	20.0–40.0	—	5.0–20.0	0.9–1.1	0.9–1.1	0.9–1.1	135.0–165.0	0.7–0.9	4.0–6.0	1.2–1.6	5.0–10.0	0.5–0.6	1.6–3.0
Nonreactive	≥ 40.0	≥ 20.0	≥ 20.0	≥ 1.1	≥ 1.1	≥ 1.1	≥ 165.0	≥ 0.9	≥ 6.0	≥ 1.6	≥ 10.0	≥ 0.6	≥ 3.0

Abbreviations: aU/mL, arbitrary units/mL; CLIA, chemiluminescence immunoassay; CMIA, chemiluminescent microparticle immunoassay; ELFA, enzyme‐linked fluorescent assay; Ig, immunoglobulin; IU/mL, international units/mL

We compared the results of the assays conducted on naïve (untreated) samples with those obtained from the same assays conducted on samples pretreated with HBT. Blocking tubes were purchased from Scantibodies Laboratory Inc. (San Diego, CA, USA). Heterophile antibodies were eliminated according to the manufacturer's instructions (Scantibodies 2009). Briefly, 500 μL of each original sample was transferred into the tube in aliquot, mixed gently by inversion, and incubated at 24°C–26°C for 1 h.

The samples were sequentially subjected to the following tests: (i) a full panel of IgM/IgG assays for EBV, HSV, VZV, CMV, rubella, and *Toxoplasma*; (ii) the same panel of assays using samples pretreated with HBT; (iii) an additional EBV assay using the Cobas e801 system (Roche Diagnostics GmbH, Mannheim, Germany) to investigate potential platform‐specific differences; and (iv) a monospot heterophile antibody test (Meridian Bioscience Inc., Cincinnati, OH, USA) using both naïve and HBT‐treated samples to confirm the presence of EBV‐specific heterophile antibodies in susptected EBV infection cases and their disappearance after HBT treatment.

The monospot test was performed according to the manufacturer's instructions. Briefly, the latex reagent and control materials were allowed to reach room temperature. The latex reagent was gently shaken to ensure even disperssion of the particles. 50 μL of the sample was pipetted onto a disposable slide. One drop of the latex reagent was then added to the sample and mixed using a stirrer. The slide was placed on a rotary shaker set at 80 rpm for 3 min. After incubation, the slide was thoroughly examined for clumping to assess the presence of agglutination. A Negative result was defined as a uniform suspension with no visible clumping, while a positive result was indicated by the presence of small to large visible clumps.

### 2.5 Patient Information and Subgroup Classification

Patient clinical data were retrieved from Severance Clinical Research Analysis Portal, a data warehouse developed and maintained by our institution. Baseline characteristics, including sex and age, were recorded. In addition, EBV quantitative PCR results were included if the blood draw for PCR was performed within ±1 day of the serologic assay. Ultimately, the patients were categorized into five subgroups based on the clinical indications for serologic testing. Those tested because of suspected acute primary viral infection, such as infectious mononucleosis, were classified as having either “suspected EBV infection” or “other suspected viral infections.” For tests related to solid organ or hematopoietic stem cell transplantation, patients were categorized into the “transplantation‐related surveillance” group if they were recipients, and into the “solid organ or peripheral blood stem cell (PBSC) donors” group if they were donors. Patients who did not fall into any of the above categories were classified as “others.”

### 2.6 Statistical Analyzes

Quantitative values outside the measurement range (indicated as “>” or “<”) were treated as the highest or lowest measurable values, respectively, in the quantitative analyzes. Assay results from untreated and HBT‐treated samples were compared using the paired *t*‐test. *p* values for changes in positivity rates following HBT pretreatment were calculated using the McNemar test. All statistical analyzes and data visualizations were performed using GraphPad Prism version 9 (GraphPad Software, La Jolla, CA, USA) and SPSS version 28 (IBM Inc., Armonk, NY, USA).

## 3 Results

### 3.1 Patient Characteristics and Initial Viral Serology Results

The baseline characteristics and initial serological results of the 185 participants are presented in Table 2. The participants were stratified based on the clinical purpose of viral serology testing. The most common indication was suspected viral infection, with suspected EBV infection being the most frequent. The second most common indication was viral surveillance in the context of transplantation, including post‐transplant monitoring of recipients for viral reactivation and pre‐transplant screening of donors for viral status.

**Table 2:** Demographics and laboratory results of study participants based on test indication.

Parameters	Suspected EBV infection		Other suspected infections		Transplantation surveillance		Solid organ or PBSC Donors		Others	
Indication for initial testing (*N* = 185)	39		72		28		20		26	
Sex, male (%)	108 (46.2)		28 (38.9)		20 (71.4)		10 (50.0)		14 (53.8)	
Age, years^a^	17.4 ± 17.3		48.1 ± 23.1		21.7 ± 21.4		40.1 ± 11.8		25.5 ± 22.4	
	Pos	Pos + Eq	Pos	Pos + Eq	Pos	Pos + Eq	Pos	Pos + Eq	Pos	Pos + Eq
EBV										
VCA IgM (%)	22 (56.4)	36 (92.3)	4 (5.6)	16 (22.2)	7 (25.0)	18 (64.3)	2 (10.0)	9 (45.0)	3 (11.5)	12 (46.2)
VCA IgG (%)	30 (76.9)	30 (76.9)	68 (94.4)	68 (94.4)	26 (92.9)	26 (92.9)	20 (100.0)	20 (100.0)	17 (65.4)	17 (65.4)
EBNA IgG (%)	18 (46.2)	22 (56.4)	68 (94.4)	68 (94.4)	17 (60.7)	20 (71.4)	19 (95.0)	19 (95.0)	17 (65.4)	19 (73.1)
HSV										
IgM (%)	20 (51.3)	25 (64.1)	41 (56.9)	56 (77.8)	9 (32.1)	10 (35.7)	9 (45.0)	12 (60.0)	13 (50.0)	17 (65.4)
IgG (%)	19 (48.7)	20 (51.3)	61 (84.7)	63 (87.5)	11 (39.3)	12 (42.9)	11 (55.0)	11 (55.0)	14 (53.8)	14 (53.8)
VZV										
IgM (%)	3 (7.7)	4 (10.3)	10 (13.9)	13 (18.1)	2 (7.1)	3 (10.7)	0 (0.0)	0 (0.0)	0 (0.0)	0 (0.0)
IgG (%)	26 (66.7)	28 (71.8)	68 (94.4)	69 (95.8)	18 (64.3)	20 (71.4)	18 (90.0)	18 (90.0)	17 (65.4)	17 (65.4)
CMV										
IgM (%)	2 (5.1)	2 (5.1)	6 (8.3)	9 (12.5)	2 (7.1)	4 (14.3)	0 (0.0)	0 (0.0)	1 (3.8)	2 (7.7)
IgG (%)	21 (53.8)	21 (53.8)	67 (93.1)	67 (93.1)	24 (85.7)	24 (85.7)	16 (80.0)	16 (80.0)	18 (69.2)	18 (69.2)
Rubella										
IgM (%)	1 (2.6)	1 (2.6)	3 (4.2)	4 (5.6)	1 (3.6)	1 (3.6)	0 (0.0)	0 (0.0)	0 (0.0)	0 (0.0)
IgG (%)	36 (92.3)	36 (92.3)	55 (76.4)	65 (90.3)	19 (67.9)	21 (75.0)	12 (60.0)	16 (80.0)	19 (73.1)	22 (84.6)
Toxoplasma										
IgM (%)	1 (2.6)	1 (2.6)	4 (5.6)	4 (5.6)	0 (0.0)	0 (0.0)	0 (0.0)	0 (0.0)	0 (0.0)	0 (0.0)
IgG (%)	2 (5.1)	2 (5.1)	5 (6.9)	5 (6.9)	2 (7.1)	2 (7.1)	1 (5.0)	1 (5.0)	2 (7.7)	2 (7.7)
EBV RQ‐PCR (*N* = 39; positive/total, %)	8/19 (42.1)		2/4 (50.0)		10/16 (62.5)		0/0 (0.0)		0/0 (0.0)	
Heterophile Ab (*N* = 137; positive/total, %)	11/29 (37.9)		4/51 (7.8)		3/25 (12.0)		0/16 (0.0)		2/16 (12.5)	

Abbreviations: Ab, antibody; CMV, cytomegalovirus; EBV, Epstein–Barr virus; EBNA, Epstein–Barr nuclear antigen; Eq, equivocal; HSV, herpes simplex virus; Ig, immunoglobulin; PBSC, peripheral blood stem cells; Pos, positive; RQ‐PCR, real‐time quantitative polymerase chain reaction; VCA, viral capsid antigen; VZV, varicella‐zoster virus.

^a^ These values are expressed as Mean ± Standard Deviation.

The positivity rates for IgM and IgG antibodies against each viral antigen varied substantially according to the clinical indication. A notable number of IgM results were interpreted as equivocal, resulting in markedly higher positivity rates when the equivocal results were considered positive. EBV VCA IgM positivity was considerably higher in patients with suspected EBV infection than in patients in other groups (56.4% vs. 11.0%, respectively). In contrast, HSV IgM positivity was consistently moderate to high across all groups, ranging from 32.1% to 56.9%.

### 3.2 Samples With Suspected False Positivity for Acute EBV Infection

**Figure 1:**
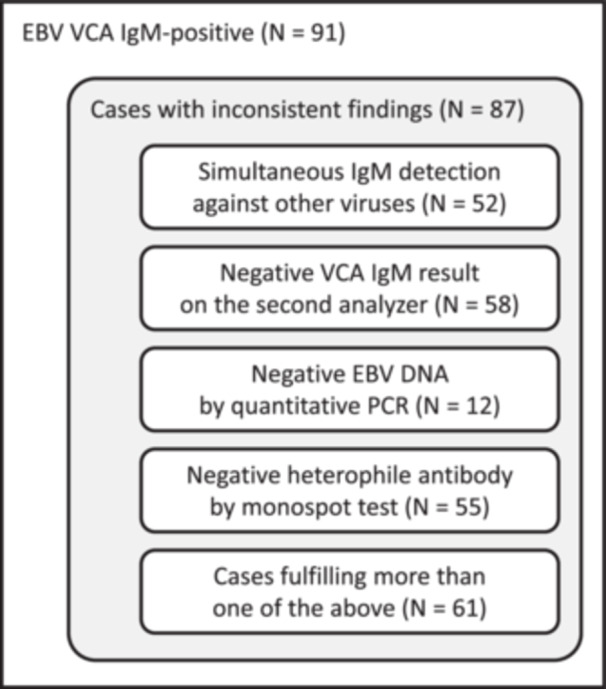
EBV VCA IgM‐positive cases with findings suggestive of false positivity.

Specifically, we aimed to identify cases with suspected false‐positive results for acute EBV infection and to analyze changes in assay results following HBT pretreatment. Interestingly, most EBV VCA IgM‐positive cases (87 out of 91) had at least one finding suggestive of potential false positivity (Figure 1): (i) concurrent IgM positivity for multiple viruses—predominantly EBV and HSV—was observed in many cases (52 out of 87); (ii) a considerable number of EBV VCA IgM‐positive samples tested negative by the Elecsys EBV assay performed on the Cobas e801 analyzer (58 out of 87); (iii) EBV quantitative PCR results were available for a subset of patients (29 out of 87), with 12 showing negative EBV DNA results; and iv) of the 71 patients who underwent the monospot test, 55 tested negative for heterophile antibodies.

### 3.3 Effect of HBT Application on Serologic Results

**Figure 2:**
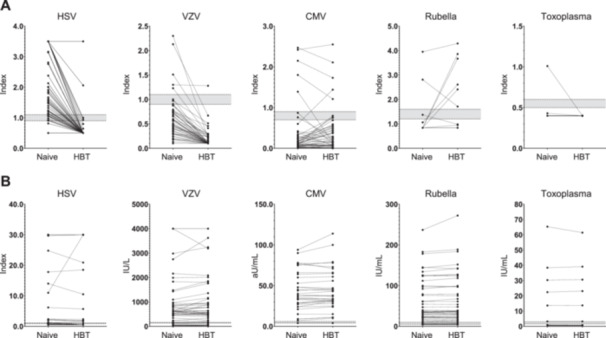
Effect of HBT pretreatment on serologic results for each virus in samples with concurrent IgM positivity for multiple viruses (*N* = 52). (A) IgM reactivity. (B) IgG reactivity. Cutoff values for each assay are indicated by horizontal dotted lines, and equivocal zones are shaded in gray.

We first compared the IgM and IgG results for various viruses other than EBV between naïve and HBT‐pretreated samples among those exhibiting concurrent IgM positivity (*N* = 52; Figure 2). HSV and CMV IgM levels showed the most pronounced reductions in reactivity following HBT pretreatment. Notably, HSV IgM exhibited a high initial positivity rate, as many results exceeded the assay cutoff, resulting in a marked decline in positivity after HBT application. In contrast, only relatively minor differences were observed in IgG reactivity between naïve and HBT‐pretreated samples.

**Figure 3:**
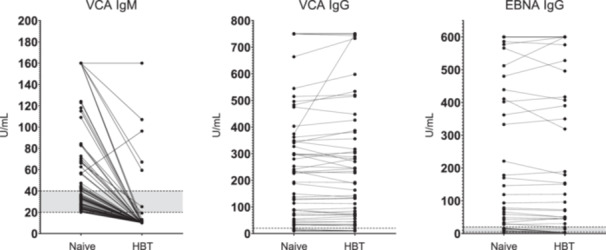
Effect of HBT treatment on EBV serologic testing in samples with possible false‐positive VCA IgM results (*N* = 87). From left to right: VCA IgM, VCA IgG, and EBNA IgG reactivity. Cutoff values for each assay are indicated by horizontal dotted lines, and equivocal zones are shaded in gray.

Next, we compared EBV serologic results between naïve and HBT‐pretreated samples suspected of having false‐positive VCA IgM results (*N* = 87; Figure 3). A dramatic reduction in VCA IgM reactivity was observed, with most samples initially classified as positive or equivocal shifting to negative. However, the effect of HBT on VCA IgG and EBNA IgG reactivity was generally limited. Table 3 summarizes the reductions in the average reactivity and positivity rates for all samples (*N* = 185) across each assay.

**Table 3:** Effect of HBT pretreatment on positivity rates and quantitative assay results.

	Naïve		HBT‐treated			
	Pos	Pos + Eq	Pos	Pos + Eq	*p* value	
EBV						
VCA IgM, U/mL	32.2 ± 35.8		12.8 ± 15.6		< 0.001	
Positivity, %	38 (20.5)	91 (49.2)	5 (2.7)	8 (4.3)	< 0.001	< 0.001
VCA IgG, U/mL	396.5 ± 303.2		402.2 ± 307.4		0.073	
Positivity, %	161 (87.0)	—	160 (86.5)	—	1.000	—
EBNA IgG, U/mL	312.8 ± 259.6		314.4 ± 261.5		0.364	
Positivity, %	139 (75.1)	148 (80.0)	138 (74.6)	140 (75.7)	1.000	0.013
HSV						
IgM, Index	1.4 ± 1.0		0.6 ± 0.4		< 0.001	
Positivity, %	92 (49.7)	120 (64.9)	5 (2.7)	7 (3.8)	< 0.001	< 0.001
IgG, Index	14.2 ± 13.8		14.2 ± 13.9		0.560	
Positivity, %	116 (62.7)	120 (64.9)	111 (60.0)	113 (61.1)	0.131	0.046
VZV						
IgM, Index	0.5 ± 0.5		0.2 ± 0.3		< 0.001	
Positivity, %	15 (8.1)	20 (10.8)	3 (1.6)	4 (2.2)	0.002	< 0.001
IgG, IU/mL	1,233.8 ± 1,167.9		1,256.3 ± 1,173.8		0.321	
Positivity, %	147 (79.5)	152 (82.2)	149 (80.5)	152 (82.2)	0.617	1.000
CMV						
IgM, Index	0.2 ± 0.4		0.3 ± 0.4		0.286	
Positivity, %	11 (5.9)	17 (9.2)	10 (5.4)	19 (10.3)	1.000	0.831
IgG, aU/mL	38.5 ± 34.6		39.9 ± 36.8		0.001	
Positivity, %	146 (78.9)	—	146 (78.9)	—	1.000	—
Rubella						
IgM, Index	0.9 ± 0.4		1.0 ± 0.6		0.008	
Positivity, %	5 (2.7)	6 (3.2)	12 (6.5)	16 (8.6)	0.023	0.009
IgG, IU/mL	46.9 ± 58.3		47.3 ± 59.0		0.141	
Positivity, %	141 (76.2)	160 (86.5)	143 (77.3)	160 (86.5)	0.480	1.000
Toxolasma						
IgM, Index	0.5 ± 0.6		0.4 ± 0.2		0.207	
Positivity, %	5 (2.7)	—	3 (1.6)	—	0.617	—
IgG, IU/mL	3.1 ± 16.3		3.0 ± 16.2		0.294	
Positivity, %	12 (6.5)	—	12 (6.5)	—	1.000	—

*Note:* Quantitative values are expressed as Mean ± Standard deviation.

Abbreviations: aU/mL, arbitrary units/mL; CMV, cytomegalovirus; EBV, Epstein–Barr virus; EBNA, Epstein–Barr nuclear antigen; Eq, equivocal; HBT, heterophile blocking tube; HSV, herpes simplex virus; Ig, immunoglobulin; IU/mL, international units/mL; Pos, positive; U/mL, units/mL; VCA, viral capsid antigen; VZV, varicella‐zoster virus.

### 3.4 Impact of HBT Application on Clinical Interpretation of EBV Status

Finally, we evaluated the clinical implications of assay result changes on the interpretation of EBV status (Table 4). The results were interpreted according to the manufacturer's recommendations (Supporting Information Table S1). The most significant changes were observed in the group with a suspected EBV infection. Initially, this group included a substantial number of patients considered to be in the early phase of the primary infection, as indicated by isolated VCA IgM positivity, or in the convalescent phase, as indicated by positivity for VCA IgM, VCA IgG, and EBNA IgG. As VCA IgM reactivity shifted to negative in many of these patients, they were reclassified as seronegative or having a past infection.

**Table 4:** Impact of HBT pretreatment on EBV status interpretation.

	Suspected EBV infection		Other suspected viral infections		Transplantation‐related surveillance		Solid organ or PBSC Donors		Others	
EBV status interpretation	Naïve	HBT‐treated	Naïve	HBT‐treated	Naïve	HBT‐treated	Naïve	HBT‐treated	Naïve	HBT‐treated
Seronegative (%)	1 (2.6)	9 (23.1)	0 (0.0)	2 (2.8)	1 (3.6)	2 (7.1)	0 (0.0)	0 (0.0)	2 (7.7)	9 (34.6)
Primary infection (%)										
(suspected early phase)	8 (20.5)	1 (2.6)	2 (2.8)	0 (0.0)	1 (3.6)	0 (0.0)	0 (0.0)	0 (0.0)	7 (26.9)	0 (0.0)
(acute phase)	12 (30.8)	4 (10.3)	0 (0.0)	0 (0.0)	8 (28.6)	1 (3.6)	0 (0.0)	0 (0.0)	0 (0.0)	0 (0.0)
(suspected convalescent phase)	7 (17.9)	0 (0.0)	3 (4.2)	0 (0.0)	2 (7.1)	1 (3.6)	2 (10.0)	0 (0.0)	1 (3.8)	0 (0.0)
Isolated VCA IgG‐positive (%)	0 (0.0)	8 (20.5)	2 (2.8)	2 (2.8)	1 (3.6)	8 (28.6)	1 (5.0)	1 (5.0)	0 (0.0)	0 (0.0)
Past infection (%)	11 (28.2)	17 (43.6)	63 (87.5)	66 (91.7)	15 (53.6)	16 (57.1)	17 (85.0)	19 (95.0)	16 (61.5)	17 (65.4)
Indeterminate (%)	0 (0.0)	0 (0.0)	2 (2.8)	2 (2.8)	0 (0.0)	0 (0.0)	0 (0.0)	0 (0.0)	0 (0.0)	0 (0.0)

Abbreviations: EBV, Epstein–Barr virus; HBT, heterophile blocking tube; Ig, immunoglobulin; PBSC, peripheral blood stem cell; VCA, viral capsid antigen.

Similarly, in the “other suspected viral infection” group, patients whose EBV status was initially interpreted as early primary infection or convalescent phase were also reclassified as either seronegative or having a past infection, following the loss of VCA IgM reactivity after HBT pretreatment.

## 4 Discussion

We frequently observed viral serological results suggestive of false reactivity in our clinical laboratory. On average, approximately 700 samples are submitted monthly for viral serological testing, with the most common indications being suspicion of acute EBV or HSV infection and post‐transplantation surveillance for viral reactivation. Because the clinical manifestations of acute viral infections are often nonspecific, serologic assays for multiple viruses—not only the most suspected pathogens—are frequently ordered concurrently as a preset panel. We observed spontaneous IgM reactivity to two or more viruses in a significant proportion of cases, which is highly improbable and raises concerns regarding assay interference. In some cases, this hypothesis was further supported by negative follow‐up PCR results. These observations provide the rationale for conducting this study.

The IgM positivity rates for HSV appeared so high that they were difficult to clinically justify (Table 2). The transplantation organ donor group can serve as a proxy for the otherwise healthy controls. In this group, when equivocal results were treated as positive, 12 of the 20 (60.0%) patients tested positive for HSV IgM. This strongly suggests a substantial number of false‐positive results. One possible explanation for the high IgM positivity rate is cross‐reactivity with VZV (Liermann et al. 2014). However, this seems unlikely in the present context, as none of the 20 individuals in the organ donor group tested positive for VZV‐IgM.

Most samples were referred for EBV serology, and the accuracy of the EBV VCA IgM results was a primary concern. Therefore, we identified and further analyzed cases with findings suggestive of false‐positive EBV VCA IgM results, although these did not constitute definitive evidence of false positivity (Figure 1). For instance, although rare, simultaneous acute infections can occur, particularly in immunosuppressed or immunocompromised individuals (Handous et al. 2020; Ito et al. 2009; Gangemi et al. 2021). A negative PCR result may indicate that the virus had already been cleared at the time of testing, even in cases of primary infection. Moreover, the sensitivity of heterophile antibody testing has been reported to be as low as 81% with certain assay kits (Bruu et al. 2000). Hence, these findings should be interpreted as suggestive rather than conclusive, indicating a high likelihood of false‐positive results in many cases, without implying that all such cases are indeed false positives.

Most samples with simultaneous IgM reactivity against multiple pathogens showed markedly reduced HSV‐ and VZV‐IgM reactivity (Figure 2 and Table 3). In contrast, some samples showed increased reactivity to CMV or rubella IgM. These findings suggest that, although all assays are based on the same sandwich immunoassay principle, heterophile antibodies may interfere in different ways depending on the structure of the target or reagent antibodies, leading to either false‐positive or false‐negative results. This observation is in line with what has been reported in the literature. Although much less frequent than false‐positive observations, false‐negative or falsely low laboratory results for clinically important analytes have also been described, such as falsely decreased total IGF‐1 or thyroglobulin levels (Brugts et al. 2009; Giovanella et al. 2009; Giovanella and Ghelfo 2007). In such cases, heterophile antibodies may not bridge the capture and detection antibodies in place of the target antigen, but instead occupy the binding sites of either the capture or detection antibody. This phenomenon is thought to occur when heterophile antibodies bind to only one of the assay antibodies—either the capture or the detection antibody—thereby preventing that antibody from binding to the antigen. Following pretreatment with HBT, a notable decline in EBV VCA IgM reactivity was observed. Not only did initially equivocal results convert to negative, but even samples that had previously tested positive or strongly positive—including those exceeding the upper limit of the reportable range (> 160 U/mL)—dropped below 20 U/mL, resulting in negative results (Figure 3 and Table 3).

Interestingly, a considerable number of samples that became nonreactive after HBT treatment had initially tested negative by the monospot assay. This discrepancy, in addition to being influenced by the relatively low sensitivity of the monospot assay, likely reflects the different nature of the antibodies targeted by the two approaches. Whereas the monospot test is designed to detect a more selective subset of heterophile antibodies associated with infectious mononucleosis from EBV infection, HBT treatment adsorbs a broader range of interfering antibodies, including those consistent with the classical definition of heterophile antibodies (antibodies that bind to animal immunoglobulins) (Lowenthal et al. 1973; Seitanidis 1969; Kricka 1999). Thus, HBT treatment may resolve interferences not captured by the monospot assay, explaining why many samples tested negative by monospot assay nevertheless demonstrated reduced reactivity after HBT treatment.

The changes in EBV VCA IgM reactivity significantly affected the final interpretation of the patients' EBV infection status. Table 4 summarizes the impact of HBT pretreatment on EBV status interpretation. In addition to suspected cases of infectious mononucleosis, EBV status misclassification has important clinical implications for transplant recipients, as post‐transplant lymphoproliferative disorder (PTLD) is a serious adverse event that can occur after transplantation (Taylor et al. 2005; Al Hamed et al. 2020). Falsely positive or equivocal VCA IgM results may give clinicians a misleading impression of EBV reactivation, and when discordant with negative EBV PCR results, may lead to unnecessary clinical concerns or confusion. Notably, in the transplantation‐related surveillance group, the most prominent finding was that most patients initially classified as having a primary infection (n = 8), were reclassified as isolated VCA IgG‐positive following conversion of VCA IgM results to negative. The isolated VCA IgG pattern has previously been observed in 7.3% of patients undergoing EBV serologic testing in outpatient settings, most commonly among adults with a past infection (De Paschale et al. 2009). EBNA IgG is not produced in approximately 5% of infected individuals and may gradually disappear in some, particularly under immunosuppressed conditions (De Paschale 2012).

This study had some limitations. First, quantitative EBV PCR results were only available for a subset of the samples. Therefore, although unlikely, the possibility of false‐negative results because of reduced reactivity of target‐specific IgM following HBT pretreatment cannot be entirely ruled out. Additionally, the limited sample volume imposed certain constraints on the study design. Ideally, the comparator assay for EBV serology‒the Elecsys EBV assay‒should also be performed separately on both naïve and HBT‐treated samples. A previous study comparing the Liaison and Elecsys assays reported a substantial discrepancy between the two (211 of 1043; 20%) (Lupo et al. 2023). The authors noted that the Elecsys assay yielded more false‐negative VCA IgM results (7 vs. 0) and had a lower sensitivity for detecting primary EBV infection (81.8% vs. 93.5%) than the Liaison assay. However, in most discordant cases, the authors used a third complementary assay—the VIDAS EBV assay—as a reference. Based on our findings, it is possible that the Liaison assay results in those discordant cases were actually false positives, rather than Elecsys results being false negatives. Additional testing using HBT‐treated samples on the Elecsys platform would help clarify whether the discrepancies observed in this study were because of heterophile antibody interference and whether the Elecsys assay is less susceptible to such interference than the Liaison assay. Future studies with more robust designs and comprehensive reference methods, such as immunoblotting and PCR, are needed to assess the analytical performance of assays from different manufacturers more definitively.

In conclusion, this study demonstrated a substantial prevalence of potentially false‐positive IgM reactivity against common viruses during routine clinical testing. Such reactivity may lead to a significant misinterpretation of a patient's viral infection status. Furthermore, we demonstrated that HBT pretreatment is an effective strategy for eliminating interference from heterophile antibodies. Given its utility, HBT pretreatment may serve as a valuable approach for resolving discrepancies when serologic results conflict with clinical or molecular findings.

## Author Contributions

**Younhee Park:** conceptualization (lead), project administration (lead), methodology (lead), data curation (lead), formal analysis (lead), visualization (lead), writing – reviewing and editing (equal). **Junhyup Song:** investigation (lead), writing – original draft preparation (lead); **Ilyoub Jeong:** investigation (supporting). **Sinyoung Kim:** writing – reviewing and editing (equal). All authors are guarantors of this study, had full access to all the study data, and take responsibility for the integrity of the data and the accuracy of the data analysis.

## Conflicts of Interest

The authors declare that they have no competing financial interests or personal relationships that may have influenced the work reported in this study.

## Supporting information

Supplemental Table 1: Interpretation of EBV assay results suggested by the manufacturer.

## Data Availability

Anonymized data are available from the authors upon request. Please contact younheep@yuhs.ac for any questions or requests.
